# Alcohol Intake and Arterial Hypertension: Retelling of a Multifaceted Story

**DOI:** 10.3390/nu15040958

**Published:** 2023-02-15

**Authors:** Antonio Vacca, Luca Bulfone, Sebastiano Cicco, Gabriele Brosolo, Andrea Da Porto, Giorgio Soardo, Cristiana Catena, Leonardo A. Sechi

**Affiliations:** 1 Clinica Medica, Department of Medicine, European Excellence Center for Arterial Hypertension, University of Udine, 33100 Udine, Italy; 2 Internal Medicine, Department of Precision and Regenerative Medicine, University of Bari, 70124 Bari, Italy

**Keywords:** alcohol withdrawal, blood pressure, cardiovascular risk factors, diastolic dysfunction, endothelial dysfunction, left ventricular hypertrophy

## Abstract

Alcoholic beverages are common components of diets worldwide and understanding their effects on humans’ health is crucial. Because hypertension is the leading risk factor for cardiovascular diseases and all-cause mortality, the relationship of alcohol consumption with blood pressure (BP) has been the subject of extensive investigation. For the purpose of this review, we searched the terms “alcohol”, “ethanol”, and “arterial hypertension” on Pubmed MeSH and selected the most relevant studies. Short-term studies showed a biphasic BP response after ingestion of high doses of alcohol, and sustained alcohol consumption above 30 g/day, significantly, and dose-dependently, increased the risk for hypertension. These untoward effects of alcoholic beverages on BP can be mediated by a multiplicity of neurohormonal mechanisms. In addition to the effects on BP, excess alcohol intake might contribute to cardiac and renal hypertensive organ damage, although some studies suggest possible benefits of moderate alcohol consumption on additional cardiovascular risk factors, such as diabetes and lipoprotein(a). Some intervention studies and cumulative analyses support the evidence of a benefit of the reduction/withdrawal of alcohol consumption on BP and cardiovascular outcomes. This is why guidelines of scientific societies recommend avoidance or limitation of alcohol intake below one unit/day for women and two units/day for men. This narrative article overviews all these topics, providing an update of the current knowledge on the relationship between alcohol and BP.

## 1. Introduction

For a very long time, consumption of alcoholic beverages has been considered an exclusive practice of humans [1]. Humans began intentional fermentation during the meso-neolithic era, and therefore, alcohol consumption has been broadly viewed as a rather recent practice, relative to the origin of the species. For this reason, it is a common tenet that the practice of alcohol consumption has had progressive and hand-in-hand development with the social and cultural evolution of humans. Alternatively and interestingly, it was hypothesized that alcohol consumption in humans might originate from evolutionary linkage, with exposure of our ancestors to sugars of ripe fruit, with the associated alcoholic fermentation by yeasts (the so called “*drunken monkey*” hypothesis) [2]. In fact, alcohol intake has always characterized all frugivorous and nectivore animals and has similarly involved primates and hominoid ancestors during the evolution of our species.

Currently, around 2.3 billion people in the world [3] ingest alcoholic drinks, and the World Health Organization Status Report on Alcohol Consumption 2019 reported the European Union (EU) as the highest in the world concerning per capita alcohol consumption [4]. Current drinking is conventionally defined as the consumption of a beverage containing alcohol, during the previous 12-month period, in the population aged 15 years and older [5]. One unit of alcohol is defined by the amount of anhydrous ethanol and, according to the World Health Organization, corresponds to 10 g of pure ethanol, although this definition is not unanimously accepted across country borders. Moreover, for the general public, alcohol consumption is more commonly estimated in amounts of wine, beer, or spirits because these are the most frequently consumed drinks. Epidemiologic data indicate that, on average, regular drinkers consume 33 g of anhydrous ethanol per day, and that beer (34%) is the most frequently ingested alcoholic drink [5].

Because of the widespread and ever-growing use of alcoholic beverages worldwide, the effects of alcohol on humans’ health have been examined in depth, and evidence indicates a strong causal association of excess alcohol consumption with severe hepatic, neuropsychiatric, metabolic disease conditions, and cancer [6]. Cardiovascular diseases are the major cause of mortality, morbidity, and disability worldwide, and excessive or binge drinking of alcoholic beverages is detrimental to cardiovascular health. US Nutrition Guidelines state that avoidance of alcoholic drinks is the best choice for health and, for people who are alcohol consumers, that daily intake should be limited to one drink or less for women or two drinks or less for men [7]. As indicated in the guidelines, one drink is equal to a 33 cl. (11 fluid ounces) bottle of regular beer, a glass of wine, a shot of liquor like rum or vodka, or a mixed drink like rum and cola [8]. Nonetheless, epidemiological evidence has emerged in support of the hypothesis of an inverse correlation between moderate consumption of alcoholic beverages and major cardiovascular events [9]. The “*French Paradox*” originated from the observation of a decreased incidence of ischemic heart disease in consumers of red wine [10], an observation that has triggered an intense and ongoing debate among nutritionists, scientists, and physicians about the potential benefits of red wine. In fact, some alcoholic beverages contain a mixture of compounds that, besides ethanol, may have beneficial results because of their antioxidant, anti-inflammatory, and cytoprotective effects [11]. Against this possibility, it was shown that the beneficial antioxidant effect of red wine components could be counterbalanced by the pro-oxidant effects of ethanol metabolites [12]. Moreover, a meta-analysis of cohort studies suggested that the cardio-protective effect of moderate drinking, reported in previous studies, could be due to selection bias [13]. Additionally, in a Mendelian randomization study that was conducted according to a variant of alcohol dehydrogenase, the protective effect of moderate alcohol intake was not observed when patients were randomized per genetic variant [14]. This finding was subsequently confirmed in another study [15].

Because of its high prevalence in the general population, arterial hypertension is considered the leading risk factor for cardiovascular diseases and all-cause mortality worldwide [16]. The prevalence of hypertension is progressively rising because of the aging of the population and an increase in exposure to unhealthy lifestyles, mainly related to dietary habits. Lifestyle interventions in hypertension require the correction of risk factors, such as overweight, sedentary life, excess sodium and low potassium dietary consumption, and smoking, but also avoidance of excessive alcohol intake [17]. Consistently, guidelines on hypertension treatment published by the International Society of Hypertension [18], European Society of Cardiology/European Society of Hypertension [19], and American Heart Association/American College of Cardiology [20], in addition to recommending weight loss for overweight/obese subjects and regular physical exercise, suggest to limit daily consumption of alcohol to no more than one drink/day for women and no more than two drinks/day for men. Because epidemiologic evidence indicates that the amount of alcoholic beverages might contribute to blood pressure increase, extensive investigations were conducted to examine the short-term and long-term effects of alcohol on blood pressure, the contribution of alcohol intake to hypertension and hypertension-related organ damage, and the effects of alcohol withdrawal in hypertensive patients. By overviewing these topics, this review aims to provide an update on the current views of the relationship between alcohol and blood pressure. For the purpose of this narrative review, we systematically searched the medical literature in English language using the Pubmed MeSH and the terms “alcohol”, “ethanol”, and “arterial hypertension” for extraction. Articles on “pulmonary arterial hypertension” were excluded. Considered publications were full-text articles, with original human or animal data for the effect of changes in alcohol intake on blood pressure levels, and meta-analyses and reviews on this same subject. A.V., L.B., and S.C. retrieved the articles that were reviewed and discussed them with L.A.S., as a fourth reviewer, for subsequent article selection. Article selection was performed according to the quality of evidence of studies that was estimated following the Grading of Recommendations, Assessment, Development, and Evaluations (GRADE) criteria that are based upon the study design, dimension, consistency, and magnitude and dose-dependency of effect [21]. Only studies rated with moderate-to-high GRADE certainty ratings were considered.

## 2. Short-Term and Long-Term Effects of Alcohol on Blood Pressure

### 2.1. Short-Term Effect of Alcohol on Blood Pressure

It is well known that the ingestion of alcoholic beverages induces systemic vasodilatory response. This could, somehow, be in contrast with the presumed chronic effects of alcohol consumption on hypertension. Placebo controlled studies of the effects of alcohol administration were performed in healthy volunteers using continuous blood pressure monitoring. An early and dose-dependent decrease in systolic and diastolic blood pressure was observed that was associated with heart rate increase, followed by a late blood pressure rebound [22]. The biphasic effect of alcohol on blood pressure was subsequently confirmed in hypertensive obese patients [23]. The short-term effects of alcohol on blood pressure were the subject of a recent systematic Cochrane review [24]. Thirty-two randomized placebo-controlled trials, including 767 participants, were analyzed, comparing the effects of blood pressure with different doses of alcohol. Ingested amounts of alcohol were standardized, according to a Canadian standard [25], as low (≤14 g of alcohol), medium (>14 g and ≤30 g of alcohol for men and >14 g and ≤20 g of alcohol for women), and high dose (>30 g of alcohol for men and >20 g of alcohol for women). At 6 h after ingestion, a low dose of alcohol did not have significant effects on blood pressure, but increased heart rate. A medium dose of alcohol decreased systolic and diastolic blood pressure by 5.6 and 4.0 mmHg, respectively, an effect that vanished at 7 h after alcohol ingestion. The high dose of alcohol induced a biphasic effect on blood pressure. After 6 h, systolic blood pressure decreased by 3.5 and diastolic blood pressure by 1.9 mmHg, changes that were maintained between 7 and 12 h after ingestion. However, more than 13 h after ingestion, the high dose of alcohol increased systolic blood pressure by 3.7 mmHg and diastolic blood pressure by 2.4 mmHg, with a significant increase in heart rate that persisted up to 24 h after ingestion. Thus, a short-term biphasic blood pressure response follows consumption of high doses of alcohol. Despite absolute short-term changes of blood pressure appearing to be quite small after alcohol ingestion, these changes could be sustained over time in chronic alcohol consumers and become relevant for chronic blood pressure control.

### 2.2. Long-Term Effect of Alcohol on Blood Pressure

Initial assessment of the effects of chronic alcohol consumption on blood pressure was provided by the Atherosclerotic Risk in Communities (ARIC) study [26]. In this prospective study, that was conducted in a multiethnic cohort, a higher risk of becoming hypertensive was independently associated with a consumption of 210 or more g of alcohol per week compared with participants who did not drink any alcoholic beverages. This study also observed a greater impact of alcohol consumption on blood pressure in Black subjects compared to White subjects, an observation that was also reported in another study conducted in a different population setting [27]. Later on, many cohort studies have confirmed the association between chronic consumption of high amounts of alcoholic beverages and incidence of hypertension [26], and most of these studies were included in comprehensive cumulative analyses and meta-analyses. Because of the apparent differences in alcohol effects on blood pressure in different ethnic groups, some meta-analyses also examined differences between Asian and Western populations. Despite methodological limitations, due to the design of studies included in this analysis, the study reported a stronger association between alcohol consumption and incident hypertension in Asian subjects compared to Western subjects [28]. The same issue was investigated in another metanalysis that showed higher sensitivity of blood pressure to alcohol intake (10 g/day) in Blacks than Whites and Asians [29]. Explanations for these possible ethnic differences to alcohol exposure, however, remain elusive.

Another relevant issue is related to possible differences of alcohol effects on blood pressure between genders. Roerecke et al., in a meta-analysis, included 20 cohort studies involving more than 350,000 subjects of both sexes, 90,160 of whom had incident hypertension [30]. When compared with abstainers, men had a relative risk for hypertension of 1.19 (C.I. 1.07–1.31) for ingestion of one to two drinks per day (corresponding to 12 g of anhydrous ethanol per drink), 1.51 (C.I. 1.30–1.76) for 3 to 4 drinks per day, and 1.74 (C.I. 1.35–2.24) for consumption of five or more drinks per day. Instead, women had no increased risk for consumption of one to two drinks per day but had an increased risk for consumption over this amount (relative risk of 1.42; C.I. 1.22–1.66), suggesting differences of blood pressure sensitivity to alcohol consumption between sexes. This difference, however, was not confirmed in another meta-analysis [29], and an absence of significant differences in sensitivity of blood pressure to chronic consumption of alcoholic beverages, between men and women, was also concluded by Puddey et al. in a thorough revision of the existing literature [31].

Thus, the current knowledge supports the view that sustained alcohol consumption, above 30 g per day, significantly, and dose-dependently, increases the risk of hypertension [32]. Differences in blood pressure sensitivity to alcohol intake among different ethnic groups, and between genders, are suggested in many studies but need further investigation. Inconsistencies among cohort studies and cumulative analyses could be due to many confounders that could not be adequately taken into account. These include the type of alcoholic beverages (beer, wine, spirits), patterns of alcohol intake (regular intake or binge drinking), variability of intake across the study periods, and possible bias in subjective measurement of alcohol consumption by use of questionnaires.

### 2.3. Neurohormonal Effects of Alcohol

A number of mechanisms, mostly related to neural and hormonal responses to alcohol consumption, might have causal involvement in alcohol-induced hypertension [33]. Activation of the sympathetic nervous system [21,34], with increased catecholamines release [35], is one of the mechanisms most frequently called into question to explain the prohypertensive action of alcohol consumption in heavy drinkers. Moreover, it was shown that alcohol modifies the response of carotid baroreceptors [36] and their participation in blood pressure regulation. Increased secretion of endorphin and histamine might mediate these effects of alcohol on baroreflex sensitivity [37].

Acute alcohol consumption activates plasma renin activity [38], increasing angiotensin II generation and stimulating aldosterone and vasopressin secretion, thereby leading to vasoconstriction and sodium and water retention [39]. Persistent increase in plasma cortisol was also reported by Jenkins et al. in heavy alcohol drinkers [40], suggesting a role of this hormone in alcohol-induced hypertension [38,41,42].

Additional experimental animal and human data suggest that alcohol consumption impairs endothelial function, decreasing nitric oxide generation and increasing formation of reactive oxygen species and oxidative stress [43]. Moreover, both acute [44] and chronic [45] ethanol exposure stimulate endothelin release from cultured human endothelial cells. All these effects of alcohol might contribute to blood pressure increase in heavy drinkers.

## 3. Does Alcohol Intake Contribute to Hypertensive Organ Damage and Cardiovascular Disease?

Separation of the effects of alcohol and hypertension on organ damage and cardiovascular disease is an arduous task, and many studies over the years have tried to assess the respective contributions.

First of all, it is important to separate the different settings, considering on one hand alcohol dependents who consume more than 90 g/day of ethanol and are at risk of developing non-ischemic dilated cardiomyopathy [46], along with many additional heart-related problems and, on the other hand, people at cardiovascular risk who consume much lower amounts of alcoholic beverages. As previously stated, the US 2020–2025 Dietary Guidelines recommend avoidance of alcohol consumption [7], and this is because no clear evidence exists to currently support possible benefits of mild to moderate alcohol consumption on cardiovascular health. Nonetheless, the hypothesis of a J-shaped relationship between alcohol intake and cardiovascular outcomes was proposed, and this opened a currently unsolved question on the definition of the amount of alcohol per week that might be considered safe or even beneficial. The definition of this threshold would be very difficult to set and, as previously stated, scientific societies in the cardiovascular field recommend no more than two alcohol units/day for men and one unit/day for women.

An important study in a metanalysis has recently tried to analyze this issue, including 83 studies from 19 high-income countries, accounting for 599,912 current consumers of alcoholic drinks [47]. The minimum risk in overall mortality among drinkers was detected with an intake around 100 g/week or below of alcohol. A positive linear relationship was observed between the amount of alcohol intake and stroke, coronary heart disease, except myocardial infarction, fatal hypertensive disease, heart failure, and fatal aortic aneurysm. Interestingly, the risk of myocardial infarction was inversely related to alcohol intake.

This matter is further complicated by the different impact that different alcoholic beverages have on cardiovascular health. For instance, the effects of spirits on the cardiovascular system might be different from those of wine because of its content of polyphenols [48,49] that are known to decrease oxidative stress and counteract inflammation [50], although some studies do not support this view [12,30].

Another aspect that deserves specific consideration is related to the so called “*masked hypertension*”, a condition involving those patients who have normal office blood pressure but are hypertensive at home [51]. This condition is very common among alcohol dependents [52,53] and carries a cardiovascular risk similar to those of patients with sustained hypertension [54].

### 3.1. Alcohol and Hypertensive Organ Damage

It is a well-established notion that consumption of high amounts of alcohol over a long period of time leads to alcohol-induced cardiomyopathy, causing heart failure, arrythmias, and atherosclerosis through multiple mechanisms [55,56,57]. On the other hand, a study by Larsson et al. [58] reported a decreased risk of heart failure in mild to moderate drinkers consuming less than 14 drinks per week. Alcohol consumption above this amount increased the risk of developing heart failure, a finding that has been confirmed in a recent study [59]. For these reasons, the European Society of Cardiology identifies excessive alcohol intake as a risk factor for heart failure and recommends no or light alcohol intake in the general population and absolute abstinence from alcohol consumption in patients with cardiac dysfunction [60].

Subclinical cardiac damage in hypertensive patients is characterized by left ventricular hypertrophy and early onset of diastolic dysfunction. Early general population studies suggested a possible association of alcohol consumption with left ventricular hypertrophy, that was more prominent in men than women, and appeared to vary according to the beverage type [61]. The same association was subsequently reported in a rural Chinese population in whom Yang et al. observed that left ventricular mass was directly related to the amount and frequency of alcohol consumption [62]. It is important to point out that, in the Yang study, this relationship was also present in a subset of patients with hypertension and was independent of blood pressure levels. A direct relationship between alcohol intake and left ventricular hypertrophy was also reported in Japanese hypertensive patients by Seki et al., and was independent from blood pressure levels and metabolic variables [63]. Interestingly, this study pointed to the possibility that the relationship between alcohol and hypertension could be somehow related to uric acid. It is well established, in fact, that an increase in uric acid could be secondary to alcohol ingestion [64,65], and previous studies reported a significant and independent association of uric acid levels with hypertension [66] and left ventricular hypertrophy [67].

Hypertension-related structural and functional left ventricular changes were investigated in 335 nonalcoholic patients with essential hypertension by use of tissue-Doppler echocardiography [68]. Patients were grouped according to different levels of daily alcohol consumption, and blood pressure levels did not differ significantly among the groups. Left ventricular hypertrophy was found in 21% of hypertensive patients and diastolic dysfunction was found in 50%. Alcohol consumption was comparable in patients with and without left ventricular hypertrophy, whereas patients with diastolic dysfunction had significantly increased daily alcohol intake. Variables indicating left ventricular diastolic dysfunction worsened progressively with increasing levels of alcohol consumption, and patients consuming 20 g/day or more of alcohol had significantly increased prevalence of diastolic dysfunction. Alcohol consumption predicted diastolic dysfunction independently of age, body mass index, blood pressure, insulin sensitivity, and left ventricular mass. Similar findings were reported in the elderly population by Gonçalves et al. [69]. Thus, current evidence indicates that alcohol consumption might affect left ventricular diastolic properties, even in nonalcoholic patients, whereas data on a possible relationship between alcohol intake and hypertrophy are limited to some ethnic groups and need further investigation.

It is well known that high blood pressure causes damage to the kidney and alcohol might add some detrimental effects to renal function in hypertension. While low amounts of alcohol appear to reduce albuminuria and slow progression of renal dysfunction [70,71], heavy alcohol consumers are exposed to the risk of serious renal complications [72]. The kidneys have some involvement in alcohol metabolism because approximately 10% of the ingested ethanol is excreted by the kidney, without any further transformation [73,74,75]. Ethanol has direct toxic effects on tubular cells [76] and increases the generation of reactive oxygen and nitrogen species that cause leukocyte infiltration and other inflammatory changes [77,78,79,80], leading to progressive impairment of renal function and albuminuria. However, a recent meta-analysis of 25 prospective cohort studies has reported that light (<12 g/day), moderate (12–24 g/day), and heavy (>24 g/day) alcohol consumption is protective against chronic kidney disease [81]. To our knowledge, there are no studies specifically designed to assess the relevance of alcohol consumption to hypertensive renal disease.

### 3.2. Type 2 Diabetes and Lipid Metabolism

Hypertension causes target organ damage in synergy with other major cardiovascular risk factors, including diabetes and dyslipidemia [82]. Therefore, effects of alcohol on these metabolic variables pose a serious threat on the cardiovascular risk of hypertensive patients.

Because of the interference of several confounders, it would be difficult to determine what could be the relevance of alcohol consumption for the lifetime risk of developing type 2 diabetes. These confounders include many factors that are associated with drinking habits, such as higher body mass index, poor dietary choices, and smoking. In a retrospective Dutch study that followed, for 10 years, 35,625 patients with a lifestyle at low risk for diabetes, moderate alcohol consumption granted 40% lower risk of new onset diabetes [83]. This study, however, clearly pointed out the need to recommend strict control over classic diabetes risk factors, rather than advocating for alcohol consumption. Similar findings were reported in another prospective study of 3042 Greek patients, of both sexes, who were followed for 10 years [84]. The risk of type 2 diabetes was evaluated in linkage with the amount of alcohol ingested, reporting a U-shaped relationship, with a 53% lower risk of diabetes in patients consuming 1 glass of wine per day than abstainers. In both the Dutch and Greek studies, multivariable adjustments for possible confounders that included body weight, physical exercise, smoking, and type of diet were completed. Nonetheless, in the Greek study, the protective effect of alcohol was further strengthened by strict adhesion to a Mediterranean diet, reiterating once again the need for appropriate dietary intervention. In a meta-analysis of 38 studies, including more than one million subjects, reductions in the risk of type 2 diabetes were found at all levels of alcohol intake of less than 63 g/day relative to teetotalers, with risk increasing above this threshold [85].

Chronic alcohol consumption is known to increase plasma levels of HDL- and LDL-cholesterol and triglycerides [86,87,88], effects that might be relevant for hypertension-related organ damage. Lipoprotein(a) is a heterogeneous lipoprotein that incorporates the highly polymorphic apolipoprotein(a). Previous studies demonstrated that serum lipoprotein(a) concentrations and apolipoprotein(a) phenotypes are strong predictors of the presence and severity of target-organ damage in patients with hypertension [89]. We investigated the relationships between alcohol consumption and serum lipoprotein(a) in 402 untreated patients, with essential hypertension, in whom the cardiovascular status was accurately assessed [90]. Light alcohol drinkers (1–20 g/day of ethanol), moderate drinkers (21–50 g/day), and heavy drinkers (>50 g/day) had, respectively, 21, 26, and 57% lower median lipoprotein(a) concentrations than occasional drinkers and teetotalers. Lipoprotein(a) levels were inversely and independently correlated with alcohol consumption in both men and women, and patients with evidence of cardiovascular damage had greater levels of lipoprotein(a) than patients without such evidence. In this study, the inverse association between alcohol intake and Lp(a) levels was independent of major confounders that included age, sex, smoking, LDL-cholesterol, fibrinogen, and glomerular filtration rate. Similar findings with evidence of an inverse relationship between alcohol intake and lipoprotein(a) concentrations was reported in 10,154 middle-aged patients [91]. It was concluded that reduction of lipoprotein(a) levels, by regular consumption of moderate amounts of alcohol, might turn beneficial for organ protection in patients with hypertension. Effects of alcoholic beverages on circulating lipoprotein(a) may therefore contribute to explaining the J-curve shaped relationship between alcohol consumption and cardiovascular disease, particularly in patients with hypertension. Additional cardiovascular benefits of mild to moderate alcohol consumption could be related to increased HDL-cholesterol and adiponectin levels [92], reduction of prothrombotic factors [93], and proinflammatory cytokines, such as C-reactive protein and interleukin-6 [94].

## 4. Effects of Alcohol Withdrawal in Hypertension

Two different clinical conditions can result from the interruption of alcohol consumption. In heavy alcohol drinkers, abrupt interruption of alcohol intake might result in an alcohol withdrawal syndrome (AWS), with a critical condition that might require hospitalization. Alternatively, chronic reduction/interruption of alcohol intake could be part of the lifestyle changes that are recommended for the achievement of better blood pressure control in hypertensive patients.

### 4.1. Alcohol Withdrawal Syndrome and Hypertension

Abrupt interruption of alcohol intake in heavy drinkers may cause the onset of symptoms that characterize the AWS [95]. Tremors, sweating, agitation, nausea, vomiting, tachycardia, and hypertension begin in alcohol abusers 6 to 24 h after the last alcohol intake [95]. Only a few patients, however, evolve to the psychotic manifestations and cardiovascular collapse that are the features of the delirium tremens (DT).

Classical studies conducted on heavy alcohol consumers admitted for detoxification, but without DT, evaluated patients for hypertension [96]. Within 72 h of admission, blood pressure ≥160/95 mm Hg was found in 33% of patients, 71% of whom had transitory elevation, whereas 29% required drug treatment. Alcohol dependents with transitory hypertension were older, drank greater amounts of alcohol, and had higher blood pressure response to the cold pressor test that was associated with higher circulating catecholamines levels compared to normotensive alcohol dependents [96]. These observations clearly point to an activated sympathetic system as a major determinant of hypertensive response in AWS.

Ceccanti et al. investigated blood pressure changes in chronic alcohol dependents on early alcohol withdrawal that were followed for 18 days [97]. At baseline, hypertension was found in 55% of patients and in 21% at the end of follow-up, suggesting the transient nature of blood pressure increase was caused by alcohol withdrawal. Persistence of hypertension could be explained either by longer-lasting alcohol effects or alcohol-independent hypertension. Other studies examined the effects of alcohol withdrawal on endothelial function in heavy alcohol consumers who were compared with alcohol dependents that did not modify their drinking habits and teetotallers [45]. Next to in vivo analysis, human endothelial cells were exposed to ethanol in vitro for 2 weeks, assessing the same variables that were measured in patients after withdrawal of alcohol exposure. Endothelin-1, nitric oxide, plasminogen activator inhibitor-1 (PAI-1), and von Willebrand factor were measured in plasma and supernatants of cultured cells as markers of endothelial function. Malondialdehyde and intracellular glutathione were evaluated as markers of oxidative stress, a key mechanism causing endothelial dysfunction. Alcohol exposure increased the levels of endothelin-1, nitric oxide, and PAI-1, and decreased those of the von Willebrand factor, both in vivo and in vitro. These changes were dose-dependent and were reversed after withdrawal. It was concluded that heavy alcohol intake affects endothelial function, with an effect that is mediated by an activated oxidative stress and is rapidly reversed after withdrawal.

In another study carried out in 14 hypertensive heavy alcohol consumers with referred alcohol intake of more than 200 g/day, who were followed for 30 days, cessation of alcohol intake caused a rapid and significant fall in systolic and diastolic blood pressure when compared to eight hypertensive heavy drinkers who refused to reduce alcohol consumption [98]. By the third day after alcohol withdrawal, blood pressure had significantly decreased, and normalization of values was obtained in most patients by the end of the study. Cessation of alcohol intake significantly decreased plasma aldosterone and cortisol levels, whereas there were no effects on active renin and 24 h fractionated urinary catecholamines values. At baseline, hypertensive heavy drinkers had significantly higher levels of plasma endothelin and PAI-1 than teetotallers. During the study, both endothelin and PAI-1 levels progressively decreased in hypertensive heavy drinkers who stopped alcohol intake but remained elevated in patients who maintained alcohol consumption. This study confirmed that hypertension is rapidly reversible in most heavy alcohol consumers, after alcohol withdrawal, and suggested, once again, an important contribution of endothelial factors to blood pressure increase in these patients. These findings were further confirmed in moderate-to-heavy drinkers in whom blood pressure fell significantly within a few weeks after alcohol withdrawal [97,99,100,101,102].

### 4.2. Treatment of Hypertension in Alcohol Withdrawal Syndrome

Complete alcohol abstinence must be recommended to all hypertensive alcohol dependents, as transient hypertension ensuing after alcohol withdrawal was found to be harmless in all our subjects [97]. Therefore, abstinence leads to a complete recovery from hypertension, in most cases. Hypertension is typically self-limited in the AWS, and drug treatments should be applied just to prevent possible urgency- or emergency-related complications [103]. In patients for whom there is concern for hypertensive urgency or emergency, full medical evaluation is indicated to identify any potential end-organ damage [103]. Otherwise, a general goal is to reduce pressure over a period of hours to days, with a target blood pressure of less than 160/100 mm Hg, with no lowering by more than 25–30% over the first 3 h [19].

Few data are available on hypertension management of patients with the AWS. Blood pressure might be difficult to control in patients with underlying treatment-resistant hypertension, and multiple medications could be needed in some cases. Drugs commonly used in the general wards are benzodiazepines and, in intensive care units (ICU), dexmedetomidine. Both drugs effectively reduce systolic and diastolic blood pressure [104], acting at the level of central nervous system and reducing the sympathetic discharge. In addition, as demonstrated in vitro, benzodiazepines induce vasodilatation by endothelium-dependent and independent mechanisms [105]. In a retrospective case series collected in ICU patients with AWS, dexmedetomidine was effective in reducing blood pressure, allowing reduction of benzodiazepine administration [106]. Other drugs that were employed in treatment of AWS were oral clonidine and captopril [103,107] or, when needed, parenteral drugs such as nitroglycerin, labetalol, urapidil, or sodium nitroprusside [19,107,108].

### 4.3. Reduction of Alcohol Intake and Blood Pressure

Evidence of a causal role of heavy alcohol intake in hypertension would receive strong support from evidence that the discontinuation of alcohol consumption lowers blood pressure. For this reason, many studies, including randomized clinical trials, examined the effect of reduction/withdrawal of alcohol intake in hypertension [19,102,103,104,105,106]. Because of important heterogeneity in participants’ characteristics, assessment of adherence to alcohol restriction, and follow-up duration, evidence of the possible benefits of alcohol withdrawal on blood pressure reduction, obtained in these studies, is rather weak. In a randomized controlled trial that enrolled 641 participants who consumed at least 10 drinks per week, the effects on blood pressure of an intervention program, based on cognitive-behavioral alcohol reduction, was examined and compared with a control group after 15 to 24 months of follow-up [109]. Subjects in the intervention group reported a significant decrease (−191 g per week) of alcohol consumption, but there was no difference in blood pressure decrease in comparison to controls. Similar results were reported by Kawano et al. who used 24 h blood pressure monitoring in a crossover-study, reporting a decrease in daytime systolic blood pressure by 3 ± 9 mm Hg with restriction of alcohol intake, but no significant change in 24 h blood pressure [110].

A meta-analysis of 36 randomized controlled trials reported an association between short-term reduction in alcohol consumption and blood pressure decrease [21]. In this metanalysis, the overall effect of alcohol reduction was a decrease of 3 mm Hg for systolic and 2 mm Hg for diastolic blood pressure. However, stratification of analysis by the amount of baseline alcohol intake showed no effect on blood pressure for participants who consumed up to two drinks per day. Conversely, blood pressure reduction was significant and progressively higher in participants who had baseline alcohol intake of three, four to five, and six or more drinks per day. In this study, a meta-regression model indicated a decrease of 0.91 mm Hg in systolic and 0.75 mm Hg in diastolic blood pressure per one drink per day. A Cochrane review was set by Acin et al. to examine the effects of interventions to reduce alcohol intake on blood pressure for at least 3 months [111]. Results of the analysis were primarily determined by the randomized controlled trial reported in reference [109]. Alcohol intake was significantly reduced by interventions in comparison to control subjects, but reduction did not result in differences in systolic and diastolic blood pressure changes when compared to controls. Furthermore, no differences were observed for overall or cardiovascular mortality and major cardiovascular events.

An alternative strategy for the reduction of alcoholic beverage intake could be the use of dealcoholized wine. In a small cross-over trial, Chiva-Blanch et al. examined the short-term (4 weeks) effects of dealcoholized wine on blood pressure, reporting a significant reduction of both systolic (−5.8 mm Hg) and diastolic (−2.3 mm Hg) blood pressure that was associated with an increase in plasma nitric oxide levels [112]. Another interesting approach aiming at reducing alcohol intake is a nurse-based behavioral approach. This approach was tested in 28 Japanese heavy drinkers in whom the nurse-based program led to a decrease in blood pressure, as assessed by 24 h monitoring, in comparison to 25 controls [113]. In this study, blood pressure targets (<135/85 mm Hg) were reached in 55.6% of subjects who reduced alcohol consumption in the intervention group, and only 16.7% in the control group.

Thus, some studies and cumulative analyses do support the hypothesis of a significant benefit for blood pressure from the reduction/withdrawal of alcoholic beverage consumption. These observations led most scientific societies to provide the current recommendations regarding alcohol consumption. Nonetheless, it should be kept in mind that this evidence comes principally from small-sized and relatively short-lasting studies, and therefore, possible long-term benefits of alcohol reduction in hypertensive patients are still debated. Further studies will be needed to provide a better understanding of the potential benefits of the reduction of alcohol intake in hypertension.

## 5. Conclusions

Arterial hypertension is considered the leading risk factor for cardiovascular diseases and all-cause mortality worldwide. Because of the widespread and ever-growing use of alcoholic beverages worldwide, the effects of alcohol on blood pressure have been in the spotlight for decades. Current evidence indicates that, despite some variability between genders and among different ethnic groups, sustained alcohol intake above 30 g per day significantly, and dose-dependently, increases the risk of hypertension. Excess alcohol intake might also contribute to the development of hypertension-related cardiac damage, independently of blood pressure. Furthermore, alcohol might affect additional cardiovascular risk factors that increase the cardiovascular risk of hypertensive patients. However, the evidence currently available in support of the possible benefits of the restriction of alcohol consumption on hypertension, and its complications, is all but conclusive and deserves further investigation.

## Data Availability

Not applicable.
